# Nanotechnology for Mitigating Biological Cross‐Contamination in Meat Processing

**DOI:** 10.1111/1750-3841.70291

**Published:** 2025-05-28

**Authors:** Camila Cristina Vieira Velloso, Juliana Arriel Torres, Caue Ribeiro

**Affiliations:** ^1^ Embrapa Instrumentation São Carlos Brazil

**Keywords:** biological cross‐contamination, food safety, nanoencapsulation, nanomaterials, shelf‐life extension

## Abstract

Foodborne contamination remains a significant challenge in the meat industry, with biological cross‐contamination threatening public health and product quality. Conventional preservation methods, such as chemical sanitizers, often suffer from limitations like toxic residues, volatilization, and the rise of microbial resistance. In this context, nanotechnology emerges as a transformative approach, leveraging the unique properties of nanomaterials, such as silver, zinc oxide, titanium dioxide, copper, and silica nanoparticles, carbon quantum dots, and nanocellulose, to enhance antimicrobial potency, antioxidant activity, and shelf‐life extension. This review critically examines nanoencapsulation systems as a key innovation, enabling the stabilization and controlled release of bioactive compounds like essential oils (e.g., carvacrol, thymol, eugenol) and flavonoids (e.g., naringenin). These systems not only prolong antimicrobial efficacy but also preserve sensory and nutritional attributes, aligning with consumer preferences for natural, safer, and minimally processed foods. Furthermore, the integration of nanomaterials into smart packaging and surface coatings demonstrates promise in real‐time contamination monitoring and pathogen suppression. However, scaling these technologies necessitates rigorous toxicological evaluations to address potential nanoparticle migration and environmental impacts. By synthesizing recent advancements, this review underscores nanotechnology's potential to redefine meat preservation paradigms while advocating for standardized regulatory frameworks to ensure safe, sustainable implementation.

## 1 Introduction

Ensuring food safety is a critical global priority with far‐reaching implications for public health and economic stability. In the specific context of meat processing, the risk of biological contamination during slaughtering, cutting, storage, and distribution represents a major pathway for pathogen transmission and the onset of foodborne illnesses. Traditionally, a range of preservation and processing techniques have been employed to mitigate microbial growth and prolong shelf life. These include salting, smoking, drying, fermentation, and thermal treatments, each with a long history of use and varying degrees of efficacy and safety.

However, despite their historical relevance, these traditional methods face increasing scrutiny. As highlighted by Kamboj et al. (2020), factors such as the formation of toxic residues, the volatilization of chemical sanitizers, and the development of microbial resistance have raised significant concerns regarding the continued reliance on conventional preservation techniques. Additionally, improper hygiene practices during food processing and handling have been implicated in a substantial proportion of foodborne outbreaks, with cross‐contamination and inadequate storage conditions being frequent contributors. Given these limitations, there is a growing demand for innovative, sustainable, and scientifically validated alternatives that can ensure microbial safety without compromising the nutritional and sensory qualities of meat products.

The susceptibility of meat to contamination stems from its nutrient‐rich composition, characterized by high moisture, protein, and lipid content, which fosters microbial proliferation at stages ranging from slaughter to retail. Beyond health risks, microbial activity accelerates spoilage, degrading sensory attributes such as texture, flavor, and aroma, thereby diminishing consumer acceptance (Nikmaram et al. 2018; Lamri et al. 2021). Consequently, the development of effective methods for controlling pathogenic microorganisms and extending the shelf life of meat is an ongoing priority within the industry.

The most common microbial contaminants in meat and meat products include bacteria such as *Pseudomonas* spp., *Listeria monocytogenes*, *Staphylococcus aureus*, *Salmonella enterica*, and *Escherichia coli*, as well as certain yeasts and fungi under specific conditions. While conventional approaches like chemical additives and heat processing have been widely adopted, growing consumer demand for minimally processed, “clean‐label” products and regulatory pressures to reduce synthetic preservatives have intensified the exploration of advanced technologies (Lisboa et al. 2024; Lamri et al. 2021; Kamboj et al. 2020). Emerging solutions, including ultraviolet (UV‐C) irradiation, ultrasound, and pulsed electric fields, show promise but often lack the multifunctionality required for comprehensive preservation (Lisboa et al. 2024; Lamri et al. 2021).

In this context, nanotechnology has emerged as a transformative paradigm, offering versatile tools to combat contamination while preserving sensory and nutritional integrity. Nanomaterials, such as silver nanoparticles (AgNPs), zinc oxide nanoparticles (ZnONPs), and titanium dioxide nanoparticles (TiO_2_NPs), leverage their high surface area‐to‐volume ratio and unique physicochemical properties to disrupt microbial membranes, inhibit biofilm formation, and neutralize free radicals (Anvar et al. 2021). Nanoencapsulation systems further enhance preservation efficacy by stabilizing volatile bioactive compounds, such as essential oils (EOs, e.g., carvacrol, thymol) and flavonoids (e.g., naringenin), enabling controlled release and prolonged antimicrobial action (Y. Wang et al. 2021; L. Zhang et al. 2023). Recent advancements in smart packaging, integrating nanomaterials with pH‐sensitive indicators or moisture‐regulating nanocellulose, exemplify the potential for real‐time quality monitoring and targeted pathogen suppression (He et al. 2023; Costa et al. 2021).

This review synthesizes contemporary research on nanotechnology‐driven strategies for meat preservation, with a focus on elucidating mechanistic pathways, evaluating practical applications, and addressing safety and scalability challenges. By critically analyzing the interplay between nanomaterial properties and preservation outcomes, this work aims to bridge the gap between laboratory innovation and industrial implementation, ultimately contributing to safer, more sustainable food systems.

## 2 Literature Search

The literature search was performed using the Web of Science Core Collection database, employing a Boolean search string designed to capture studies published between January 2020 and September 2024. The search strategy combined terms related to nanotechnology, including nanoparticle, nanosphere, nanocapsule, nanomaterials, and nanocomposite, with the keyword “meat” to target articles focused explicitly on meat preservation. Titles, abstracts, and keywords were screened to ensure relevance to the topic.

In the first phase of screening, articles were excluded based on titles and abstracts that did not align with the review's scope, such as studies on nonmeat food products, nonpreservation applications of nanomaterials (e.g., medical or environmental uses), or methodologies unrelated to nanotechnology. The remaining articles underwent a full‐text evaluation to confirm their suitability. Publications were excluded if they lacked experimental data specific to meat preservation, focused solely on synthetic preservatives without nanomaterial integration, or were published in non‐English languages or non‐peer‐reviewed formats.

A total of 70 articles met the final inclusion criteria and were subjected to detailed qualitative synthesis. Data extraction emphasized the study meat processing stage, nanomaterial types (metallic nanoparticles, nanocellulose, nanoencapsulated bioactive compounds), target preservation challenges (e.g., microbial contamination, lipid oxidation), and outcomes related to shelf‐life extension and sensory quality retention. To ensure objectivity and minimize bias, two independent researchers conducted the screening and data extraction processes, with discrepancies resolved through discussion and consensus.

## 3 Application of Nanoparticles in Biological Control in Meat Processing

Biological contamination in meat processing, particularly by pathogens such as *E. coli*, *Salmonella* spp., and *S. aureus*, remains a persistent challenge, driving the need for advanced interventions. Nanotechnology has emerged as a transformative tool, offering solutions that surpass conventional methods like refrigeration and chemical preservatives in efficacy and sustainability. These innovations are particularly critical in mitigating foodborne illness outbreaks linked to meat products.

Recent advancements, as summarized in Table 1, highlight diverse applications of nanostructures across meat processing stages. Most implementations occur during postprocessing conservation, typically under refrigerated conditions (4–10°C), though studies have explored broader temperature ranges. Applications span from short‐term interventions (hours) to extended preservation periods (up to 28 days). Notable strategies include nanoparticle‐infused coatings for cutting boards and additives in poultry drinking water, both demonstrating significant reductions in microbial load.

**TABLE 1:** Application of nanostructures in pathogen control at different stages of meat processing.

Meat type	Processing stage	Nanostructure	Application method	Other formulation components	Pathogens evaluated	Reference
Bovine	The film was applied in the postprocessing conservation stage (4°C for up to 5 days).	CNFs	Film	Chitosan, starch, and CEO	*S. aureus* and *E. coli*	Sreekanth et al. 2024
Bovine	The film was applied in the postprocessing conservation stage (4°C for up to 7 days).	Biological iron sulfide nanoparticles	Film	Chitosan	*S. typhimurium* and *S. aureus*	Shen et al. 2023
Bovine	The powder was applied in the postprocessing conservation stage (4°C for up to 9 days).	ChNPs	Powder	Chitosan	*E. coli* and *S. aureus*	Abdelhady et al. 2023
Bovine	The film was applied in the postprocessing conservation stage (4°C for up to 5 days).	AgNPs coated with silica	Film	PVA	*E. coli* and *S. aureus*	Zhao et al. 2022
Bovine	The coating was applied in the postprocessing conservation stage using a two‐step immersion process, followed by a drying period (4°C for up to 15 days).	ChNPs	Coating	Whey protein, sodium alginate, nisin, and CEO	Total bacteria and volatile base‐forming microorganisms	M. Zhang et al. 2022
Bovine	Absorbent pads were applied in the postprocessing conservation stage underneath the meat (4°C for up to 7 days).	AgNPs	Absorbent pads	CMC, bacterial cellulose, and citric acid	*L. monocytogenes*, *S. aureus*, *E. coli*, and *Salmonella*	Yang et al. 2022
Bovine	The film was applied in the postprocessing conservation stage (4°C for up to 14 days).	CuNPs	Film	Fish skin gelatin, chickpea protein isolate, and microencapsulated *Nigella sativa* EO	*E. coli* and *S. aureus*	Rasul et al. 2022
Bovine	The film was applied in the postprocessing conservation stage (4°C for up to 8 days).	AgNP‐ion‐doped organomodified halloysite and MMT nanoclays	Film	PCL	*S. aureus*, *L. monocytogenes*, *E. coli*, and *S. typhimurium*	İlaslan et al. 2022
Bovine	The film was applied in the postprocessing conservation stage (4°C for up to 13 days).	AgNPs–kaolinite nanoparticles	Film	Gelatin	*E. coli*, *S. enteritidis*, *S. infantis*, *Pseudomonas* spp., and *S. aureus*	Nur Hanani et al. 2022
Bovine	The coating was applied in the postprocessing conservation stage using a 60‐minute immersion process, followed by a drying period (4°C for up to 5 days).	Naringenin nanoparticles	Coating	PVA	*Bacillus cereus*, *S. aureus*, methicillin‐resistant *S. aureus*, *S. typhi*, and *Yersinia enterocolitica*	Ab Rashid et al. 2022
Bovine	The film was applied in the postprocessing conservation stage (4°C for up to 15 days).	MMT nanoclay	Film	Chitosan and ginger EO	*E. coli* and *S. aureus*, total mesophilic aerobic bacteria	Y.‐P. Zhang et al. 2021
Bovine	The film was applied in the postprocessing conservation stage (25°C for up to 18 h).	Aluminum oxide nanostructures	Aluminum foil	–	*E. coli* K‐12	Smith et al. 2021
Bovine	The film was applied in the postprocessing conservation stage (4°C for up to 15 days).	ZnONPs	Film	Grape seed extract and CMC	*E. coli* and *L. monocytogenes*	Priyadarshi et al. 2021
Bovine	The film was applied in the postprocessing conservation stage (4°C for up to 10 days).	TiO_2_NPs	Film	Chitosan and *Cymbopogon citratus* EO	*S. aureus*, *Enterobacteriaceae*, psychrotrophic bacteria, and lactic acid bacteria	Hosseinzadeh et al. 2020
Bovine	The powder was applied in the postprocessing conservation stage (4°C for up to 10 days).	ChNPs	Powder	Monoterpenes (limonene, linalool, menthol, and thymol)	*E. coli* and *S. typhimurium*	Badawy et al. 2020
Red meat	The film was applied in the postprocessing conservation stage (4°C for up to 10 days).	ChNF and Co‐MOF	Film	PVA and anthocyanins from barberry	*E. coli*, *S. aureus*, and *P. fluorescens*	Noori et al. 2024
Porcine	Nanofibers were applied to meat packaging prior to dielectric barrier cold plasma treatment and subsequently stored (4°C for up to 8 days).	SiO_2_NPs	Nanofibers	PCL and d‐cysteine	*E. coli*, *S. aureus*, *L. monocytogenes*, and *S. enteritidis*	Ye et al. 2025
Porcine	The film was applied in the postprocessing conservation stage (4°C for up to 15 days).	TiO_2_NPs	Film	Chitosan and PVA	*S. myotis*, *Acinetobacter pulli*, and *P. paralactis*	D. Wang et al. 2024
Porcine	The film was applied in the postprocessing conservation stage (25°C for up to 24 h and 4°C for up to 5 days).	ZnONPs	Film	PLA and PVA	*S. aureus* and *E. coli*	Duan et al. 2022
Porcine	The film was applied in the postprocessing conservation stage (4°C for up to 10 days).	Zein–arabic gum nanoparticles	Film	Sodium alginate and oregano EO	*S. aureus* and *E. coli*	Y. Cao et al. 2024
Porcine	The coating was applied in the postprocessing conservation stage using a 30‐minute immersion process, followed by a drying period (4°C for up to 20 days).	Ovalbumin nanoparticles	Coating	Carvacrol	*Salmonella*	R. Zhang et al. 2023
Porcine	The coating was applied in the postprocessing conservation stage using an immersion process, followed by a drying period (4°C for up to 9 days).	Carvacrol nanoemulsion	Coating	Lecithin, casein, and chitosan	*S. aureus* and *L. monocytogenes*	Zaharioudakis et al. 2023
Porcine	Absorbent pads were applied in the postprocessing conservation stage underneath the meat (4°C for up to 10 days).	Oxidized bacterial nanocellulose	Absorbent pads	–	*S. aureus* and *E. coli*.	Tian et al. 2023
Porcine	The film was applied in the postprocessing conservation stage (20°C for up to 2 days).	NP‐CDs	Film	Chitosan, starch, and GT	*L. monocytogenes*, *E. coli*, and *S. aureus*	Khan, Ezati, et al. 2023b
Porcine	The film was applied in the postprocessing conservation stage (20°C for up to 2 days).	GT‐CDs	Film	Chitosan and gelatin	*E. coli* and *L. monocytogenes*	Khan, Ezati, et al. 2023a
Porcine	Absorbent pads were applied in the postprocessing conservation stage underneath the meat (4°C for up to 5 days).	AgNPs	Absorbent pads	PVA, paper fiber, and purple sweet potato anthocyanins	*Salmonella*	He et al. 2023
Porcine	The film was applied in the postprocessing conservation stage (25°C for up to 72 h and 4°C for up to 4 days).	TiO_2_NPs	Film	Sodium alginate, chitosan, and anchoring anthocyanins	*E. coli* and *S. aureus*	S. Cao et al. 2023
Porcine	The film was applied in the postprocessing conservation stage (10°C for up to 8 days).	Copper‐modified zinc oxide nanoparticles and CNFs	Film	Gelatin, agar, and clove EO	*L. monocytogenes* and *E. coli*.	Roy et al. 2022
Porcine	Absorbent pads were applied in the postprocessing conservation stage underneath the meat (4°C for up to 14 days).	CuNPs	Absorbent pads	Chitosan and dialdehyde starch	*L. monocytogenes*, *E. coli*, *S. typhimurium*, and *S. aureus*	Chen et al. 2022
Porcine	The film was applied in the postprocessing conservation stage (10°C for up to 8 days).	ZnONPs	Film	Propolis, pullulan, and chitosan	*E. coli* and *L. monocytogenes*	Roy et al. 2021
Porcine	The coating was applied in the postprocessing conservation stage using a 3‐min immersion process, followed by a drying period (4°C for up to 14 days).	Eugenol nanocapsules	Coating	Gelatin and chitosan	Unspecified—total number of colonies (TBC)	Q. Wang et al. 2020
Poultry (chicken) and porcine	The nanofibers were applied in the postprocessing conservation stage (4°C for up to 5 days).	Gliadin nanoparticles and glycyrrhiza polysaccharide nanofibers	Nanofibers	Gum arabic and tea tree EO	*S. typhimurium*	Cai et al. 2021
Poultry (chicken)	The coating was applied in the postprocessing conservation stage using an immersion process, followed by a drying period (7°C for up to 7 days).	Thymol nanoemulsion	Coating	Chitosan	*S. enteritidis*	da Silva et al. 2024
Poultry (chicken) and caprine (goats)	The film was applied in the postprocessing conservation stage (4°C for up to 28 days).	AgNPs	Film	PLA and cellulose	*E. coli*, *P. aeruginosa*, *Enterobacter aerogenes*, and *S. aureus*	Rejiniemon et al. 2024
Poultry (chicken)	The film was applied in the postprocessing conservation stage (4°C for up to 5 days).	Quercetin‐loaded nanoliposomes	Film	Pectin, chitosan, and soy lecithin	*E. coli, L. monocytogenes*, and *S. enterica*	S. Ali et al. 2023
Poultry (chicken)	The film was applied in the postprocessing conservation stage (4°C for up to 5 days).	Eugenol‐loaded sodium caseinate and trimethyl ChNPs	Film	Gelatin	*S. aureus*	Lin, Mei, et al. 2023
Poultry (chicken) and bovine	The film was applied in the postprocessing conservation stage (4°C for up to 5 days).	SiO_2_NPs loaded with caffeic acid	Film	Cassava starch and CMC	*E. coli* and *S. aureus*	Lin, Peng, et al. 2023
Poultry (chicken) and bovine	The film was applied in the postprocessing conservation stage (4°C for up to 12 days).	Pomegranate extract‐loaded nanoparticles	Film	CMC, chitosan, and sodium alginate	*S. typhimurium*, *C. jejuni*, *S. aureus*, and *L. monocytogenes*	Khalid et al. 2024
Poultry (chicken)	The film was applied in the postprocessing conservation stage (5°C for up to 15 days).	ZnONPs	Film	Pectin	Aerobic mesophilic, aerobic psychrotrophic microorganisms, and *Enterobacteriaceae*	Przybyszewska et al. 2023
Poultry (chicken)	The film was applied in the postprocessing conservation stage (−20°C for up to 15 days and 4°C for up to 10 days).	AgNPs	Film	Starch and poly(butylene adipate co‐terephthalate)	*E. coli*, *S. aureus*, *S. enteritidis*, and *S. typhimurium*	das Neves et al. 2023
Poultry (chicken) and ovine (lamb)	The film was applied in the postprocessing conservation stage (4°C for up to 9 days [chicken] and 4°C for up to 12 days [lamb]).	ZnONPs	Film	*Artemisia sphaerocephala* Krasch gum and forsythia EO	*S. aureus* and *E. coli*	Khan, Shu, et al. 2023
Poultry (chicken)	The powder was applied in the postprocessing conservation stage (4°C for up to 4 days).	ZnONPs	Powder	Zeolite and *Aloe vera* gel	*S. typhi* and *S. paratyphi* A	Soltan Dallal et al. 2023
Poultry (chicken)	The powder was applied in the postprocessing conservation stage (4°C for up to 5 days).	AgNPs	Powder	–	*S. typhimurium* and *S. enteritidis*	Alsammarraie et al. 2023
Poultry (chicken)	The nanoparticles were added to the chickens’ drinking water for 35 days.	AgNPs	Powder	Acetic acid	*P. oryzihabitans* and *S. paucimobilis*	El‐Abd et al. 2022
Poultry (chicken)	The film was applied in the postprocessing conservation stage (4°C for up to 20 days).	Copper ion exchanged natural zeolite nanoparticles	Film	PLA	Psychrotrophic, aerobic mesophilic bacteria, Enterobacteriaceae, and *Salmonella* spp.	Vergara‐Figueroa et al. 2022
Poultry (chicken)	The film was applied in the postprocessing conservation stage (8°C for up to 5 days).	Clove EO‐loaded ChNPs–ZnONPs	Film	Pullulan and chitosan	*P. aeruginosa*, *S. aureus*, and *E. coli*	Gasti et al. 2022
Poultry (chicken)	Absorbent pads were applied in the postprocessing conservation stage underneath the meat (4°C for up to 14 days).	CNC	Absorbent pads	Chitosan	*S. aureus*, *E. coli*, and *Candida albicans*.	Costa et al. 2021
Poultry (chicken)	The film was applied in the postprocessing conservation stage (4°C for up to 8 days).	CNFs	Film	Inulin and CMC	*L. monocytogenes*, *S. aureus*, *B cereus*, *E. coli*, *S. typhimurium*, *Klebsiella pneumoniae*, *Y. enterocolitica*, *Proteus vulgaris*, *P. aeruginosa*	Zabihollahi et al. 2020
Poultry (chicken)	The film was applied in the postprocessing conservation stage (4°C for up to 12 days).	CNFs and ZnONPs	Film	Gelatin	*S. aureus* and *P. fluorescens*	Ahmadi et al. 2020
Poultry (chicken)	The film was applied in the postprocessing conservation stage (5°C for up to 11 days).	ZnONPs	Film	Chitosan	*E. coli* and *S. aureus*	Souza et al. 2020
Poultry (turkey)	The film was applied in the postprocessing conservation stage (3°C for up to 12 days).	AgNPs	Film	–	*E. coli*	Deus et al. 2017
Poultry (duck)	The film was applied in the postprocessing conservation stage (4°C for up to 15 days).	Bacterial cellulose nanofibrils and black pepper EO nanoemulsion	Film	Gelatin	*E. coli* and *S. aureus*	Acharya et al. 2024
Poultry (quail)	The film was applied in the postprocessing conservation stage (4°C for up to 12 days).	ZrO_2_NPs	Film	Starch, pectin, and *Zataria multiflora* EO	*E. coli* and *B. cereus*	Sani et al. 2021
Ovine (sheep)	The coating was applied in the postprocessing conservation stage (4°C for up to 12 days).	ZnONPs	Coating	Acetic acid	*L. monocytogenes*, *E. coli*, *S. aureus*, and *B. cereus*	Mirhosseini and Arjmand 2014
Ovine (lamb)	The film was applied in the postprocessing conservation stage (4°C for up to 9 days).	Bacterial nanocellulose	Film	Valerian root extract and karaya gum	*S. aureus* and *E. coli*	Khan, Li, et al. 2024
Ovine (lamb)	The indicator was applied to the packaging during meat storage, demonstrating its efficiency in detecting spoilage based on the formation of volatile nitrogen compounds.	CuONPs	Film	Carboxymethyl chitosan and saffron petal anthocyanin	*S. aureus* and *E. coli*	Fathi et al. 2022
Ovine (lamb)	The film was applied in the postprocessing conservation stage (4°C for up to 11 days).	ChNPs	Film	PLA and *Polylophium involucratum* EO	Total aerobic mesophilic bacteria, psychrotrophic bacteria, *Pseudomonas* spp., lactic acid bacteria, and *Enterobacteriaceae*	Tabatabaee Bafroee et al. 2020
Rabbit	The film was applied in the postprocessing conservation stage (4°C for up to 16 days).	AgNPs	Film	Berry wax and chitosan	*E. coli* and *S. aureus*	Khan, Hashim, et al. 2024
Camel	The coating was applied in the postprocessing conservation stage (4°C for up to 24 days).	ZnONPs	Coating	–	*E. coli* O157 and *L. monocytogenes*	Gharehyakheh 2024
Camel	The coating was applied in the postprocessing conservation stage (4°C for up to 20 days).	AuNPs	Coating	–	*E. coli* O157 and *L. monocytogenes*	Gharehyakheh et al. 2020
Camel	The film was applied in the postprocessing conservation stage (4°C for up to 14 days).	MMT nanoclay	Coating	Chitosan, CMC, *Ziziphora clinopodioides* EO, and *Ficus carica* extract	*L. monocytogenes* and *E. coli* O157:H7	Khezrian and Shahbazi 2018
Fish	The coating was applied in the postprocessing conservation stage using a two‐step immersion process, followed by a drying period (4°C for up to 7 days).	SeNPs	Coating	Chitosan and propolis	*E. coli*, *S. aureus*, and *S. typhimurium*	Youssef et al. 2022
Fish	The film was applied in the postprocessing conservation stage (4°C for up to 16 days).	ZnONPs	Film	PLA, *Zataria multiflora* EO, and *Mentha piperita* EO	*E. coli*, *S. enterica*, *P. aeruginosa*, *B. cereus*, and *S. aureus*.	Heydari‐Majd et al. 2019
Seafood (shrimp)	The film was applied in the postprocessing conservation stage (25°C for up to 36 h).	Co‐MOF	Film	Sodium alginate	*E. coli* and *S. aureus*	Feng et al. 2023
Seafood (shrimp)	The coating was applied in the postprocessing conservation stage using an immersion process, followed by a drying period (4°C for up to 10 days).	ChNPs	Coating	Clove extract	*E. coli*, *S. typhimurium*, and *S. aureus*	Tayel et al. 2020
Not specified	Nanoparticles can be applied to surfaces of meat processing equipment, such as cutting boards.	FeNPs	Coating	Polytetrafluoroethylene	*E. coli*	Serov et al. 2022
Not specified	The film was applied in the postprocessing conservation stage (18°C for up to 9 days).	CaO_2_NPs	Film	Polyvinylpyrrolidone, CMC, and egg albumin	*E. coli* and *S. aureus*	Gan et al. 2024

Nanostructures are predominantly applied as films (61.2%) and coatings (19.4%), leveraging their ability to form barriers against microbial adhesion and oxidative damage. Powders and absorbent pads, though less common (7.5% each), show promise in targeted antimicrobial delivery and moisture regulation. Emerging methods, such as nanofibers and aluminum foil modifications, remain underexplored but offer advantages in stability.

Meat type significantly influences nanomaterial selection and application. Poultry (28.4%), bovine (25.4%), and porcine (23.9%) products dominate research, reflecting their global consumption rates and susceptibility to spoilage. Studies on ovine (lamb, 6.0%), camel (4.5%), and aquatic species (shrimp, fish; 3% each) remain limited but underscore the adaptability of nanotechnology to diverse matrices. Other varieties include turkey, quail, sheep, rabbit, goat, and duck.

**FIGURE 1:**
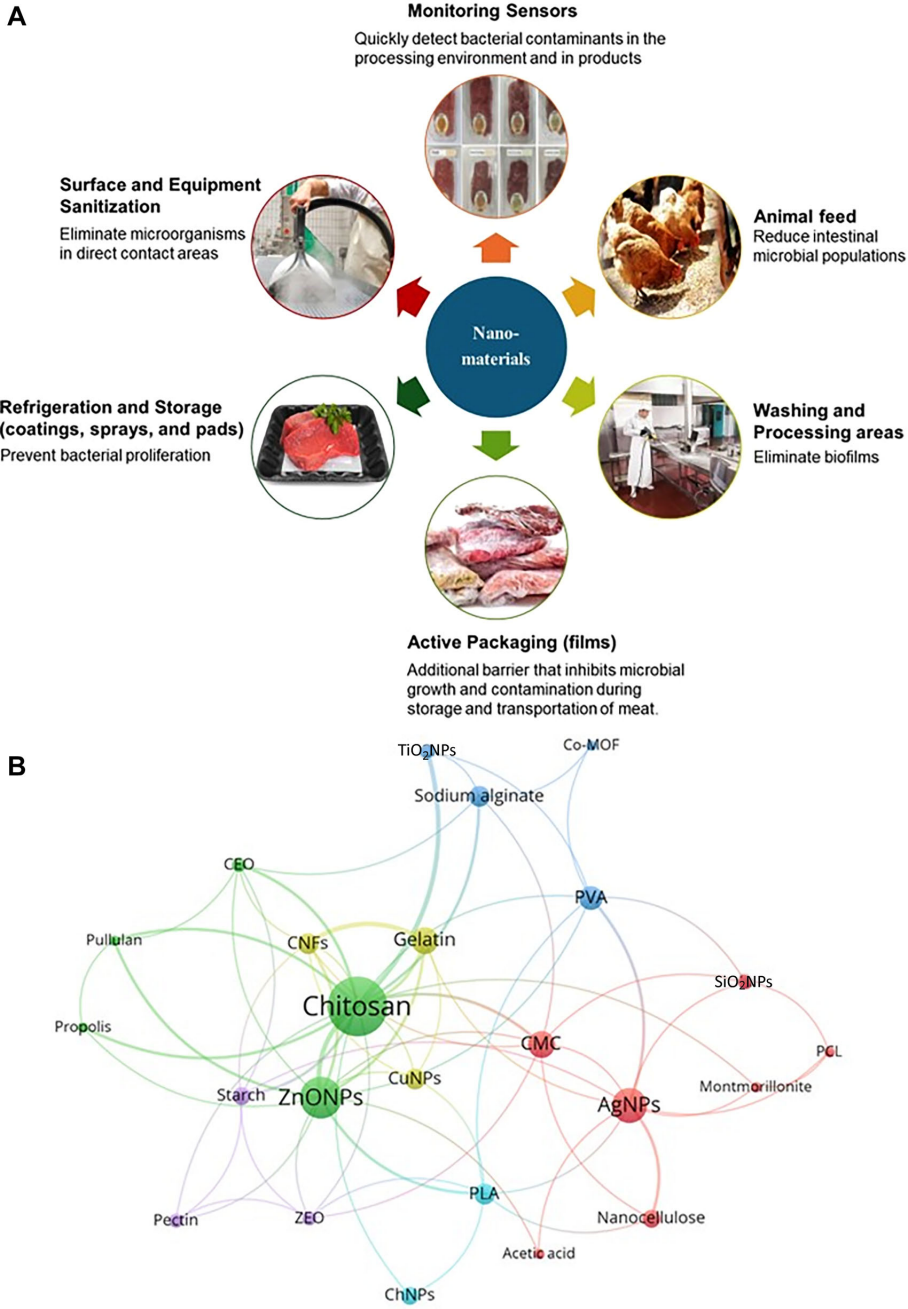
Overview of nanomaterials in the meat industry. (A) Possible applications of nanomaterials in various stages of meat processing. (B) Network analysis depicting the relationships between materials used in the meat industry, based on co‐occurrence in at least two articles from the dataset. Node size reflects the frequency of material usage, while arrows and edge thickness indicate co‐occurrence strength. Color‐coded groups denote associations calculated using the association strength method (parameters: attraction = 7; repulsion = −1). CNFs: cellulose nanofibers; PCL: polycaprolactone; PLA: poly(lactic acid); PVA: polyvinyl alcohol; ChNPs: chitosan nanoparticles; Co‐MOF: cobalt metal–organic framework nanoparticles; ZEO: essential oils of *Zataria multiflora*; CMC: carboxymethyl cellulose; CEO: cinnamon essential oil.

ZnONPs (17.9%) and AgNPs (16.4%) are the most widely studied, owing to their broad‐spectrum antimicrobial activity and regulatory acceptance. Chitosan and cellulose‐based nanomaterials (11.9%) are favored for their biodegradability and synergistic effects with bioactive compounds like EOs. Nanoclays (halloysite, montmorillonite [MMT]) and copper nanoparticles (CuNPs; 4.5%) enhance mechanical and barrier properties in packaging, while less common materials, such as cobalt metal–organic frameworks (Co‐MOFs), silica nanoparticles (SiO_2_NPs), and selenium nanoparticles (SeNPs; 1.5%–3.0%), highlight niche applications in freshness monitoring and antioxidant delivery. Figure 1 illustrates the potential applications of nanomaterials in the meat industry, emphasizing the most utilized materials and highlighting frequently employed combinations.

Pathogen specificity remains a focus, with *E. coli* and *S. aureus* tested in 85% of studies due to their prevalence in contamination events. *Salmonella* spp. (particularly *S. enteritidis* and *S. typhimurium*) and *L. monocytogenes* are also frequently evaluated, reflecting their public health significance. Less common targets, including *Bacillus* sp., *Campylobacter jejuni*, and lactic acid bacteria, validate the broad‐spectrum potential of nanomaterials but highlight gaps in understanding strain‐specific interactions.

The following topics provide a critical overview of recent advances, challenges, and future perspectives for applications of each specific nanomaterial or nanoencapsulation process in meat preservation. They highlight the ongoing efforts to enhance food safety and shelf life through targeted nanotechnology solutions. Future efforts must prioritize standardized protocols, long‐term toxicological assessments, and consumer acceptance studies to translate laboratory innovations into market‐ready solutions.

## 4 Metallic Nanoparticles

The application of metallic nanoparticles in meat preservation has emerged as a multifunctional strategy, combining antimicrobial action, oxidative control, and smart functionalities. ZnONPs and AgNPs dominate research due to their well‐documented efficacy against key pathogens such as *E. coli* and *S. aureus*, while TiO_2_NPs leverage photocatalytic properties for UV‐driven microbial inactivation. Beyond these, other metallic nanoparticles, including CuNPs, gold nanoparticles (AuNPs), iron nanoparticles (FeNPs), SeNPs, and calcium peroxide nanoparticles (CaO_2_NPs), have gained traction for innovative mechanisms such as controlled ion release, reactive oxygen species (ROS) generation, and real‐time freshness monitoring. This section critically examines these materials’ roles in enhancing microbiological safety, extending shelf life, and preserving sensory quality while addressing recent advancements, regulatory challenges, and prospects for scalable industrial adoption.

### 4.1 ZnONPs

ZnONPs have gained significant attention due to their high antimicrobial activity, antioxidant properties, and potential to improve the structural integrity of food packaging. The physicochemical properties of ZnONPs, including morphology, size, and surface reactivity, are critically influenced by synthesis methods, whether physical, chemical, or biological, and experimental parameters such as temperature and pH (Mohammadi and Ghasemi 2018). Green biosynthesis routes, utilizing microorganisms like *Aspergillus niger* and *Fusarium keratoplasticum*, have gained prominence for producing ZnONPs with diverse geometries (e.g., rods, hexagons) while minimizing environmental impact (Mohamed et al. 2019).

In meat preservation, ZnONPs exhibit robust efficacy against key pathogens. For instance, 19.7‐nm ZnONPs synthesized from *Anethum graveolens* leaf extract reduced *E. coli* and *L. monocytogenes* populations by 3–4 log CFU/g in camel meat, concurrently delaying lipid and protein oxidation to extend shelf life by 20 days under refrigeration (Gharehyakheh 2024). Similarly, ultrasonically synthesized ZnONPs demonstrated broad‐spectrum activity against Gram‐positive (*S. aureus*) and Gram‐negative (*S. enterica*) bacteria, with inhibition zones reaching 22 mm at 200 µg/mL (Nazoori and Kariminik 2018). Comparative studies further revealed that ZnO_2_NPs outperformed conventional ZnONPs in inhibiting multidrug‐resistant *S. aureus* biofilms, underscoring the role of oxidation state in antimicrobial potency (S. S. Ali et al. 2021).

The integration of ZnONPs into nanocomposite matrices has revolutionized active packaging systems. Carboxymethyl cellulose (CMC) films infused with ZnONPs and grape seed extract eradicated *E. coli* within 12 h and reduced lipid oxidation in beef by 88% over 15 days (Priyadarshi et al. 2021). Poly(lactic acid) (PLA)‐based films embedded with ZnONPs suppressed methicillin‐resistant *S. aureus* and *E. coli* by > 90%, extending pork shelf life by 120 h (Duan et al. 2022). Hybrid systems, such as pullulan–chitosan films reinforced with mushroom‐mediated ZnONPs and propolis, further enhanced UV‐blocking and mechanical properties while inhibiting *L. monocytogenes* and *E. coli* (Roy et al. 2021). These advancements are complemented by studies demonstrating ZnONPs’ efficacy in poultry preservation, where nanocomposite films reduced *Enterobacteriaceae* and *Salmonella* counts, prolonging refrigerated chicken shelf life by 11–15 days (Przybyszewska et al. 2023; Souza et al. 2020; Soltan Dallal et al. 2023).

Synergistic combinations of ZnONPs with EOs have amplified preservation outcomes. Gelatin–agar films containing copper‐doped ZnONPs and clove EO inhibited *L. monocytogenes*, reduced *E. coli* counts by 50%, and delayed lipid oxidation in pork. Similarly, *Artemisia sphaerocephala* gum films with ZnONPs and forsythia EO achieved inhibition zones of 27.1 mm against *S. aureus*, extending the shelf life of chicken and lamb (Roy et al. 2022; Khan, Shu, et al. 2023). Such hybrid systems also extended the shelf life of fish fillets from 8 to 16 days by curbing *Pseudomonas* spp. and *Salmonella* growth (Heydari‐Majd et al. 2019).

**FIGURE 2:**
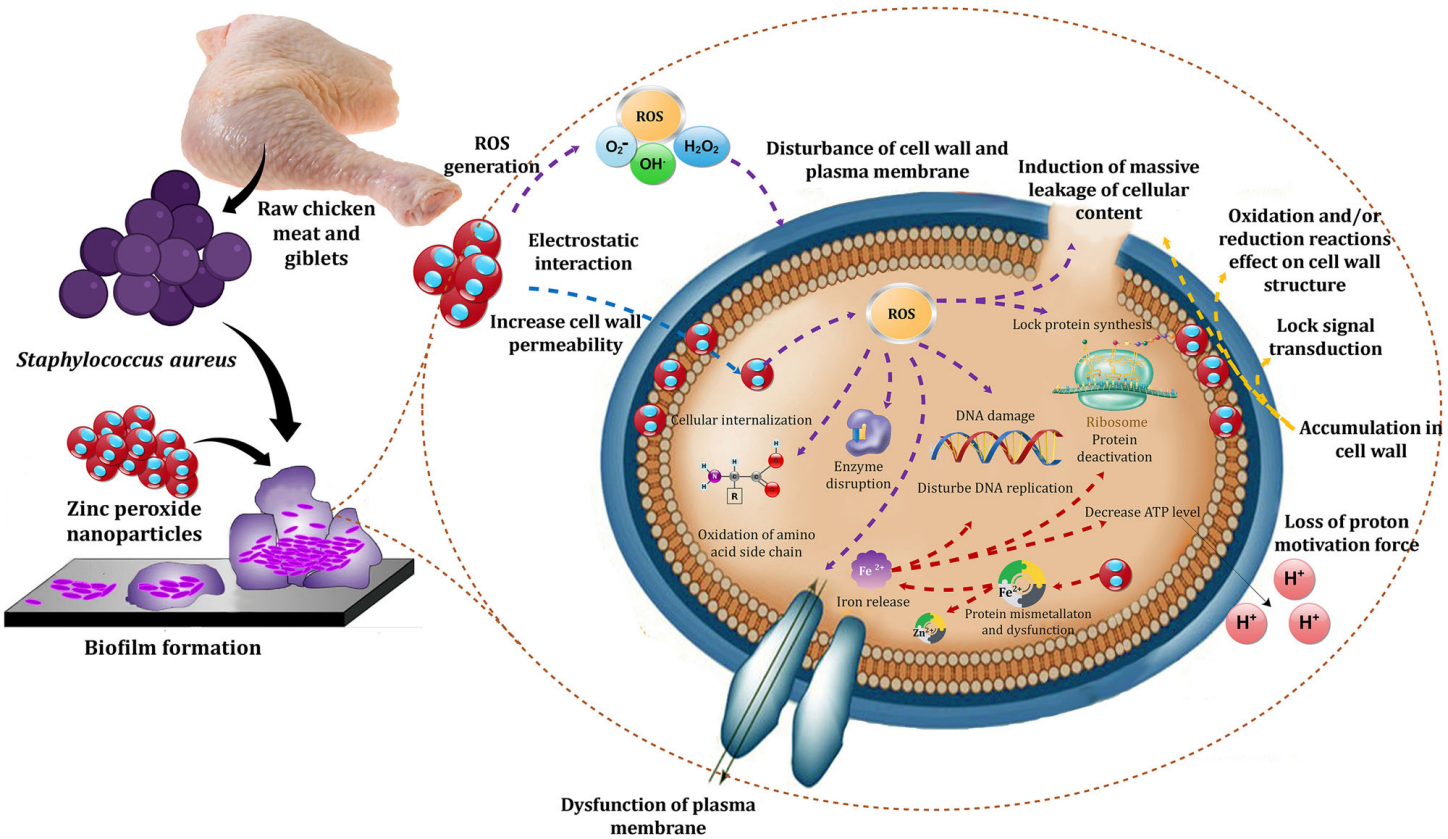
Potential mechanisms underlying the antimicrobial action of zinc nanoparticles. Reprinted with permission (S. S. Ali et al. 2021). ROS: reactive oxygen species.

The antimicrobial mechanism of ZnONPs is multifaceted (Figure 2). Nanoparticles adhere to microbial membranes, disrupting structural integrity through Zn^2+^ ion release and ROS‐mediated oxidative stress (Agarwal et al. 2018). Particle morphology significantly influences efficacy: rod‐shaped ZnONPs, with their high surface‐to‐volume ratio, exhibit superior activity compared to hexagonal variants (Mohamed et al. 2019). The synthesis method also plays a pivotal role, with biologically synthesized ZnONPs often proving more effective against fungi than chemically synthesized versions (Jamdagni et al. 2024).

Despite their GRAS (Generally Recognized as Safe) status in the United States and regulatory approval in China, concerns persist over long‐term toxicity, particularly regarding Zn^2+^ migration into fatty tissues (Ma et al. 2021). Future research must prioritize green synthesis optimization, dose standardization, and comprehensive toxicological assessments to ensure safe, scalable integration into meat processing workflows. By addressing these challenges, ZnONPs can solidify their role as sustainable, multifunctional tools in next‐generation food preservation.

### 4.2 AgNPs

AgNPs are widely acknowledged for their antimicrobial properties, which arise from the synergistic effects of metallic silver's inherent biocidal activity and the unique physicochemical attributes of nanomaterials. Their high surface‐area‐to‐volume ratio enhances interactions with bacterial cells, enabling biofilm inhibition and broad‐spectrum activity against foodborne pathogens (Franci et al. 2015). Gram‐negative (G–) bacteria, such as *E. coli* and *Salmonella* spp., exhibit greater susceptibility to AgNPs compared to Gram‐positive (G+) bacteria like *S. aureus*, owing to structural differences in cell wall composition (Donga and Chanda 2021).

Biosynthesis methods have gained traction for producing AgNPs with enhanced biocompatibility and reduced environmental impact. For instance, 47.3‐nm AgNPs synthesized using *Forsythia suspensa* extract demonstrated strong bacteriostatic activity against pathogens such as *L. monocytogenes* and *C. jejuni* (Du et al. 2019). Similarly, mango seed‐derived AgNPs (26.85 nm) showed higher efficacy against *E. coli* (G–) than *S. aureus* (G+), attributed to the thicker peptidoglycan layer in G+ bacteria (Donga and Chanda 2021). These findings underscore the influence of synthesis routes and bacterial morphology on antimicrobial performance (Zhao et al. 2022).

In meat processing, AgNPs have been integrated into diverse matrices to enhance preservation outcomes. Turmeric‐synthesized AgNPs reduced *S. enteritidis* and *S. typhimurium* contamination in poultry by 4 log CFU/g, extending shelf life by 3 weeks (Alsammarraie et al. 2023). Similarly, chitosan–waxberry films incorporating AgNPs prolonged rabbit meat preservation by 16 days, maintaining sensory quality (Khan, Hashim, et al. 2024). Innovative packaging systems, such as polyvinyl alcohol (PVA) aerogels with silica‐coated AgNPs, achieved a 5‐log reduction in *E. coli* within 6 h, though efficacy against *S. aureus* was comparatively lower due to cell wall robustness (Zhao et al. 2022).

Hybrid systems combining AgNPs with nanoclays or natural polymers further amplify functionality. Biodegradable polycaprolactone (PCL) films reinforced with halloysite‐doped AgNPs reduced bacterial loads in beef by 90% over 8 days of refrigerated storage (İlaslan et al. 2022). Similarly, gelatin films intercalated with kaolinite‐supported AgNPs suppressed *E. coli* and *S. enteritidis* in cold‐stored beef, demonstrating the potential of nanocomposites to enhance barrier properties and antimicrobial activity (Nur Hanani et al. 2022).

**FIGURE 3:**
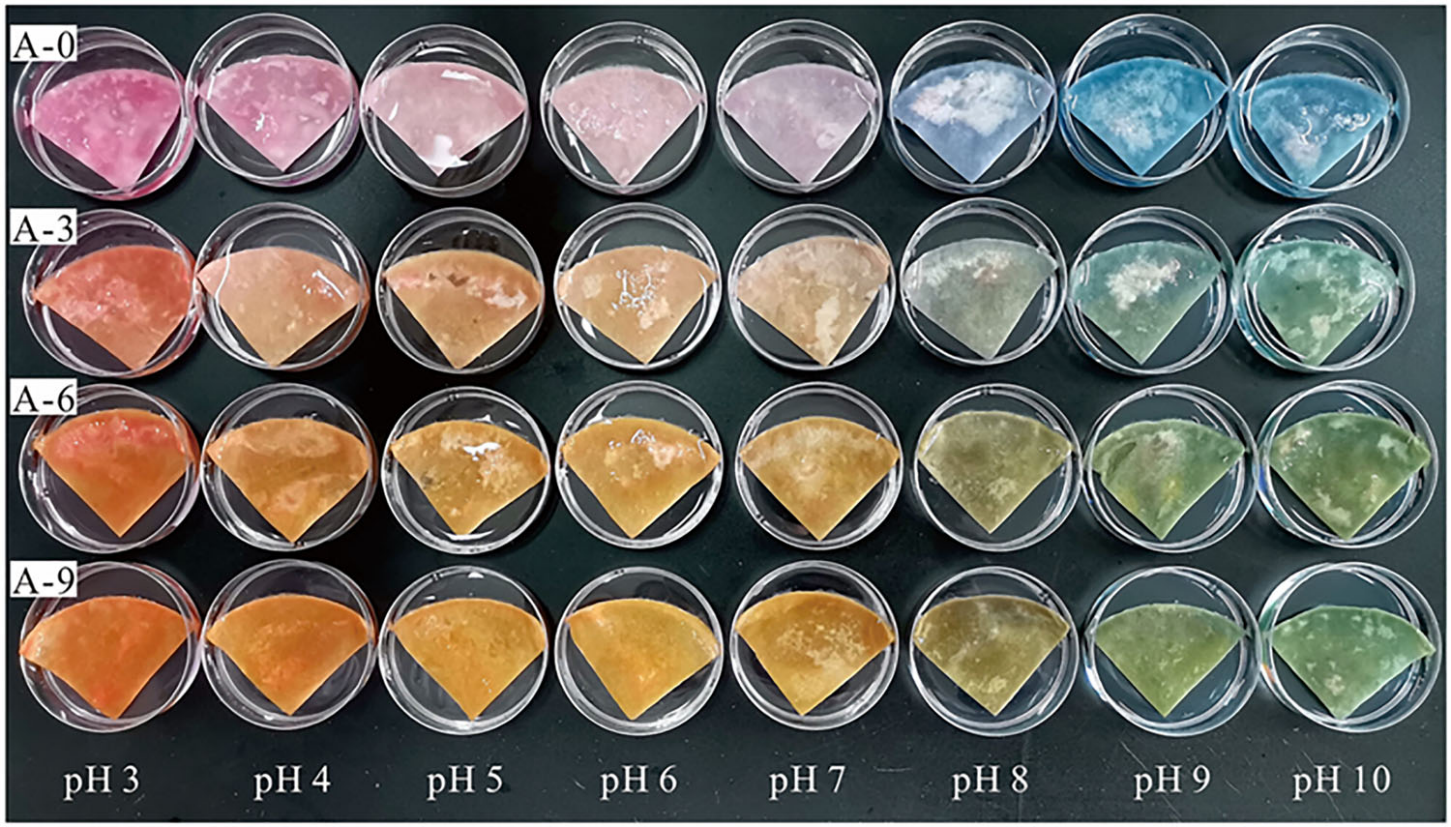
Visual changes observed in absorbent pads immersed in buffer solutions at different pH levels. Reprinted with permission (He et al. 2023).

A notable advancement is the development of smart packaging with dual functionality. PVA aerogels embedded with AgNPs and purple sweet potato anthocyanins (Figure 3) extended pork shelf life from 72 to 120 h while serving as pH‐responsive freshness indicators, transitioning from pink/red (pH 5) to blue/green (pH 9) as spoilage progressed (He et al. 2023). CMC aerogels incorporating in situ synthesized AgNPs similarly inhibited *Pseudomonas* spp. in beef, highlighting their utility in real‐time quality monitoring (Yang et al. 2022).

Beyond packaging, AgNPs have been explored in animal feed to reduce contamination. Broiler chickens administered red algae (*Corallina elongata*)‐derived AgNPs exhibited a 3‐log reduction in intestinal *Pseudomonas oryzihabitans* and *Sphingomonas paucimobilis*, without detectable silver residues in meat, suggesting a safe route for preemptive pathogen control (El‐Abd et al. 2022).

The antimicrobial effectiveness of AgNPs is also greatly influenced by factors like size, shape, and concentration. Rai et al. (2009) demonstrated that truncated triangular AgNPs needed only 1 µg to inhibit bacterial growth. In contrast, spherical particles required 12.5 µg, and rod‐shaped particles needed between 50 and 100 µg, highlighting the critical role of nanoparticle morphology. In another study, Qamer et al. (2021) reported that spherical AgNPs demonstrated higher bacteriostatic activity than rod‐shaped or linear nanoparticles at similar diameters. Although the precise mechanism of AgNPs action is not fully understood, studies suggest that silver ions interact with cell membrane proteins, enzymes, and bacterial DNA, generating ROS that induce cell death (Kalwar and Shan 2018).

**FIGURE 4:**
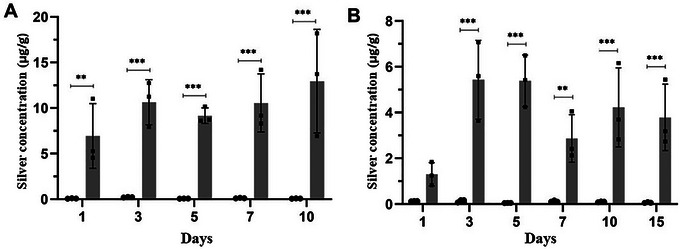
Silver migration from bio‐AgNP films to chicken meat stored under different conditions: (A) refrigerator and (B) freezer. Data are presented as mean ± standard deviation from three replicates. Statistical differences between the control (black) and bio‐AgNP film‐treated (gray) groups were analyzed using an unpaired Student's *t*‐test, with **p < 0.01 and ***p < 0.001 (das Neves et al. 2023). Reprint under the terms of the Creative Commons BY License.

Despite their promise, AgNPs pose potential risks. Long‐term exposure may impact the human immune system, particularly natural killer cell activity, necessitating stringent dose regulation (Qamer et al. 2021). Migration studies revealed silver accumulation in chicken meat exceeding permissible limits (16.98 µg/mL) when stored in bio‐AgNP films, underscoring the need for formulation refinements (Figure 4) (das Neves et al. 2023). Current safety thresholds, such as 1 ppm AgNPs (50 nm size, 11 m^2^/g surface area), are derived from toxicological models but require validation in long‐term human studies (Rezvani et al. 2019).

In conclusion, AgNPs represent a versatile tool for mitigating biological contamination in meat processing, offering rapid pathogen suppression and innovative packaging solutions. However, their safe deployment demands optimized synthesis methods, dose standardization, and rigorous migration controls to balance efficacy with consumer safety. Future research should prioritize eco‐friendly fabrication techniques and interdisciplinary studies to address regulatory and scalability challenges.

### 4.3 TiO_2_NPs

TiO_2_NPs have emerged as a prominent tool in food preservation, particularly for meat products, due to their dual antimicrobial and photocatalytic properties. These nanoparticles are increasingly integrated into packaging films and coatings to inhibit pathogenic microorganisms and extend shelf life. For instance, chitosan and PVA films embedded with TiO_2_NPs effectively suppressed spoilage pathogens such as *Serratia myotis* and *Pseudomonas paralactis* in refrigerated pork, extending preservation by 15 days through enhanced water vapor barrier properties and synergistic antimicrobial activity (D. Wang et al. 2024). Similarly, sodium alginate‐based smart films incorporating TiO_2_NPs and anthocyanins demonstrated dual functionality: they reduced *E. coli* and *S. aureus* populations by 4 log CFU/g while providing real‐time freshness monitoring via pH‐responsive color changes (S. Cao et al. 2023). These results highlight TiO_2_NPs’ potential for improving both safety and quality control in meat storage.

**FIGURE 5:**
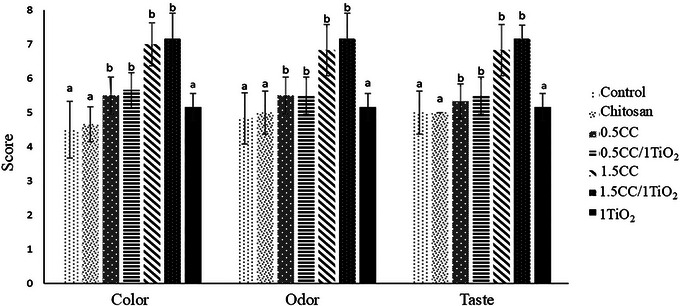
Organoleptic properties of minced meat packaged in chitosan film with *C. citratus* essential oil and TiO_2_ nanoparticles (NPs) stored at 4°C on the 4th day. Treatments include 0.5CC = 0.5% *C. citratus* essential oil; 0.5CC/1TiO_2_ = 0.5% *C. citratus* essential oil + 1% TiO_2_NPs; 1.5CC = 1.5% *C. citratus* essential oil; 1.5CC/1TiO_2_ = 1.5% *C. citratus* + 1% TiO_2_NPs; 1TiO_2_ = 1% TiO_2_NPs. Different lowercase letters indicate significant differences between treatments for each evaluated property (*p* < 0.05). Reprinted with permission (Hosseinzadeh et al. 2020).

Further advancements include chitosan films combined with 1% TiO_2_NPs and *Cymbopogon citratus* EO, which reduced microbial cross‐contamination in ground beef by 90% over 10 days of refrigeration (Hosseinzadeh et al. 2020). Notably, the incorporation of *C. citratus* EO significantly enhanced organoleptic properties (color, taste, odor) of minced meat (*p* < 0.05), while TiO_2_NPs alone showed no adverse effects on sensory quality (*p* > 0.05) (Figure 5). These findings underscore the compatibility of TiO_2_NPs with bioactive compounds in multifunctional packaging systems.

Despite these benefits, the European Food Safety Authority (EFSA) re‐evaluated titanium dioxide (E171) in 2021, concluding that TiO_2_NPs could no longer be considered safe as food additives due to concerns over genotoxicity and nanoparticle accumulation in tissues (Younes et al. 2021). This regulatory shift emphasizes the need for cautious application and further toxicological studies. Current research must prioritize the development of safer TiO_2_NPs formulations, such as surface‐modified or composite nanoparticles, to mitigate health risks while retaining antimicrobial efficacy.

In conclusion, TiO_2_NPs offer significant potential for enhancing meat preservation through pathogen inhibition and smart packaging solutions. However, their sustainable integration into food systems demands rigorous safety assessments, alignment with evolving regulatory standards, and innovative approaches to balance functionality with consumer health considerations. Future efforts should explore eco‐friendly synthesis methods and long‐term biosafety profiles to unlock their full potential in the food industry.

### 4.4 Other Metallic Nanoparticles

Beyond AgNPs, ZnONPs, and TiO_2_NPs, a diverse array of metallic nanoparticles has demonstrated significant potential in controlling biological contamination and enhancing meat preservation. CuNPs, for instance, exhibit robust antimicrobial activity due to their capacity to disrupt microbial membranes and interfere with enzymatic processes. Chen et al. (2022) developed chitosan‐based aerogels containing liposome‐encapsulated CuNPs, which reduced *L. monocytogenes*, *E. coli*, *S. typhimurium*, and *S. aureus* populations by 4–5 log CFU/g in pork, extending shelf life by 14 days. Similarly, CuNPs embedded in anthocyanin‐doped films functioned as dual‐purpose freshness indicators and antimicrobial barriers, inhibiting *S. aureus* and *E. coli* while transitioning from pink to blue as pH increased during spoilage (Fathi et al. 2022). The integration of copper‐ion‐exchanged zeolite nanoparticles into PLA films further suppressed psychrophilic and mesophilic bacteria in chicken meat without altering sensory attributes, underscoring copper's versatility in active packaging (Vergara‐Figueroa et al. 2022).

**FIGURE 6:**
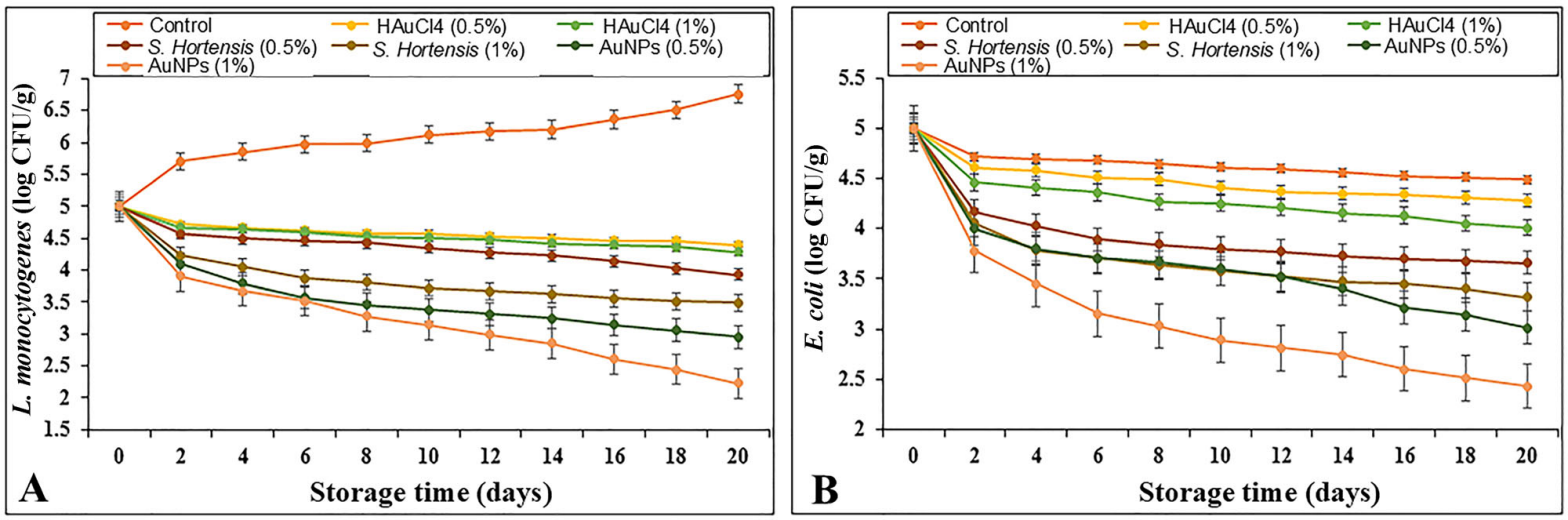
Changes in (A) *L. monocytogenes* and (B) *E. coli* populations in meat samples treated with HAuCl_4_, *S. hortensis*, and AuNPs over 20 days. Reprinted with permission (Gharehyakheh et al. 2020).

AuNPs, though less commonly studied, have shown promise in combating oxidative and microbial spoilage. *Satureja hortensis*‐synthesized AuNPs (Figure 6) reduced *L. monocytogenes* and *E. coli* populations in ground camel meat by 3 log CFU/g over 20 days, concurrently lowering thiobarbituric acid‐reactive substances (TBARS) by 40% to enhance oxidative stability (Gharehyakheh et al. 2020). Co‐MOFs represent another innovative approach, enabling real‐time spoilage detection through colorimetric responses to volatile amines. Films incorporating Co‐MOF‐encapsulated anthocyanins shifted from blue to green in the presence of ammonia, achieving simultaneous *Pseudomonas fluorescens* inhibition and freshness monitoring in beef (Noori et al. 2024; Feng et al. 2023).

**FIGURE 7:**
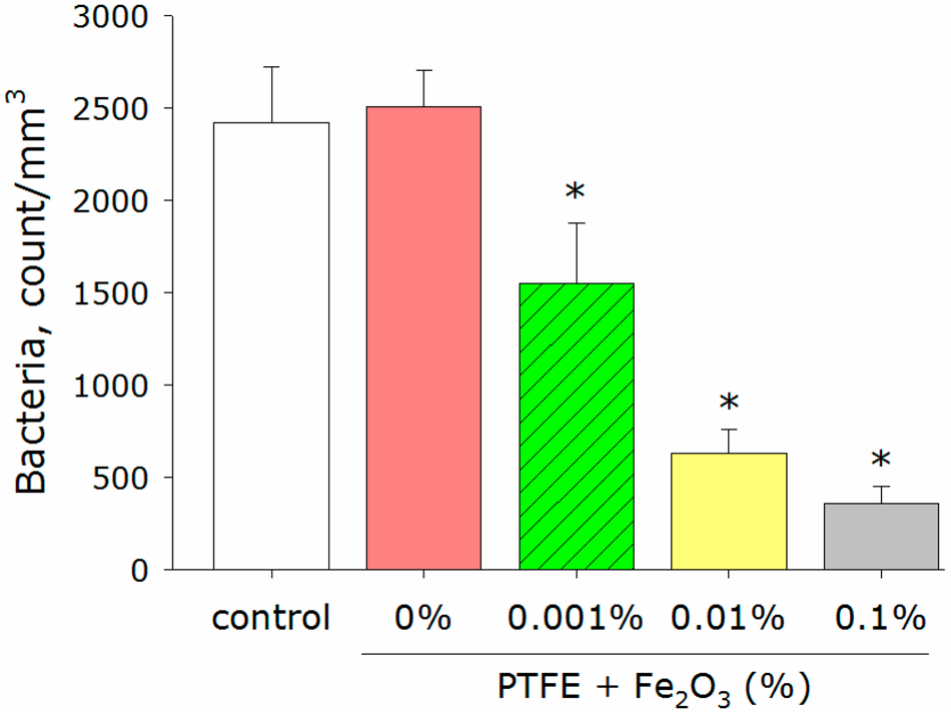
Antibacterial activity of composite PTFE/Fe_2_O_3_ nanoparticles against *E. coli*. Data are shown as means ± standard errors. *p* < 0.05 vs. control, Mann–Whitney test (*n* = 3) (Serov et al. 2022). Reprint under the terms of the Creative Commons BY License. PTFE: polytetrafluoroethylene.

**FIGURE 8:**
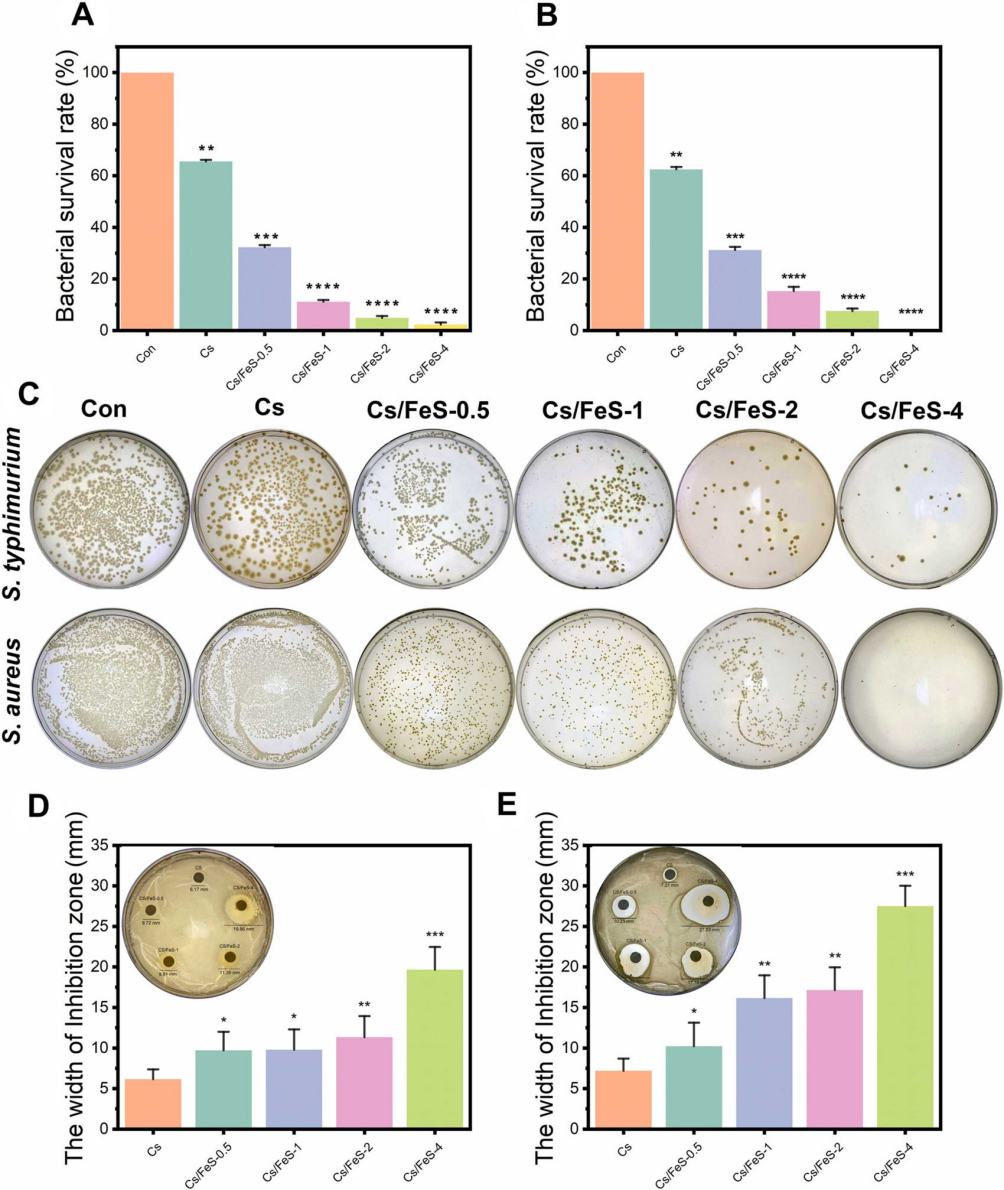
Antibacterial activity of chitosan films against *S. typhimurium* (A) and *S. aureus* (B). Images of bacterial colonies treated with chitosan films (Cs/FeS) (C). Inhibition zone size for *S. typhimurium* (D) and *S. aureus* (E). Statistically significant differences are indicated by asterisks (*p < 0.05, **p < 0.01, ***p < 0.001). Reprinted with permission (Shen et al. 2023).

Aluminum oxide nanoparticles, often overlooked in food applications, demonstrated remarkable bactericidal efficacy when nanostructured foils were applied to contaminated beef. Heat treatment (95°C, 30 min) with these foils achieved 100% inactivation of *E. coli* via ROS‐mediated membrane disruption (Smith et al. 2021). Iron‐based nanoparticles have also gained traction, particularly in surface coatings. Polytetrafluoroethylene films embedded with Fe_2_O_3_ nanoparticles reduced *E. coli* adhesion on cutting boards by 85% (Figure 7). In comparison, chitosan films functionalized with biological iron sulfide nanoparticles inactivated 99% of *S. aureus* in beef through sulfhydryl group targeting (Figure 8) (Serov et al. 2022; Shen et al. 2023).

SeNPs and CaO_2_NPs further exemplify the breadth of metallic nanomaterial applications. SeNPs incorporated into chitosan–propolis nanocomposites delayed spoilage in refrigerated catfish fillets by 7 days, suppressing *S. typhimurium* and *S. aureus* via glutathione peroxidase‐mimetic activity (Youssef et al. 2022). CaO_2_NPs, embedded in polysaccharide–protein films, achieved > 97% inactivation of *E. coli* and *S. aureus* through controlled ROS release without compromising meat texture or flavor (Gan et al. 2024).

Despite their efficacy, the commercial adoption of these nanoparticles hinges on resolving critical challenges. Long‐term toxicity studies, particularly for Co‐MOFs and aluminum oxide nanoparticles, remain sparse, while scalability issues plague selenium‐ and gold‐based systems due to high synthesis costs. Environmental concerns, such as nanoparticle migration into ecosystems, further necessitate lifecycle assessments and greener fabrication methods. Addressing these barriers through interdisciplinary research will be pivotal to harnessing the full potential of metallic nanoparticles in sustainable meat preservation.

## 5 Nonmetallic Nanoparticles

Nonmetallic nanoparticles represent a transformative frontier in meat preservation, offering eco‐friendly and biocompatible alternatives. Carbon quantum dots (CQDs), with their tunable surface chemistry and heteroatomic doping capabilities, provide dual antimicrobial and antioxidant functionalities while enabling real‐time freshness monitoring. SiO_2_NPs, prized for their porous architecture, excel in the controlled release of bioactive compounds, synergizing with advanced technologies like cold plasma to enhance preservation efficiency. Nanocellulose, derived from renewable biomass, combines mechanical robustness with pathogen‐inhibiting properties, serving as a sustainable platform for active packaging and absorbent systems. These materials address critical challenges in microbial control, oxidative stability, and sensory preservation, aligning with global demands for green and consumer‐safe solutions. This section delves into their unique mechanisms, applications, and pathways toward sustainable adoption.

### 5.1 CQDs

CQDs have emerged as versatile nano‐bacteriostatic agents in the food industry, offering a unique combination of antimicrobial efficacy, biocompatibility, and safety. These carbon‐based nanomaterials are synthesized via two primary approaches: “bottom‐up” methods, which assemble CQDs from molecular precursors (e.g., citric acid, amino acids), and “top‐down” methods, which fragment larger carbon structures (e.g., graphene, carbon nanotubes) into nanoscale particles. Techniques such as acid oxidation, hydrothermal synthesis, and microwave‐assisted pyrolysis dominate production, with postsynthesis purification (e.g., dialysis, centrifugation) ensuring uniform size distribution and enhanced functionality (Khan, Ezati, et al. 2023b; Xu et al. 2004; Wu et al. 2021).

CQDs exhibit broad‐spectrum antimicrobial activity against G+, G−, and fungal species, attributed to their tunable surface chemistry and charge‐mediated interactions. Heteroatomic doping, particularly with nitrogen (N‐CQDs) or sulfur (S‐CQDs), enhances their reactivity by introducing functional groups that disrupt microbial membranes. For instance, N‐CQDs leverage amine and amide groups to electrostatically bind to negatively charged bacterial surfaces, while S‐CQDs generate sulfonic acid moieties that degrade cell integrity (Ghirardello et al. 2021; Jhonsi et al. 2018; Travlou et al. 2018).

**FIGURE 9:**
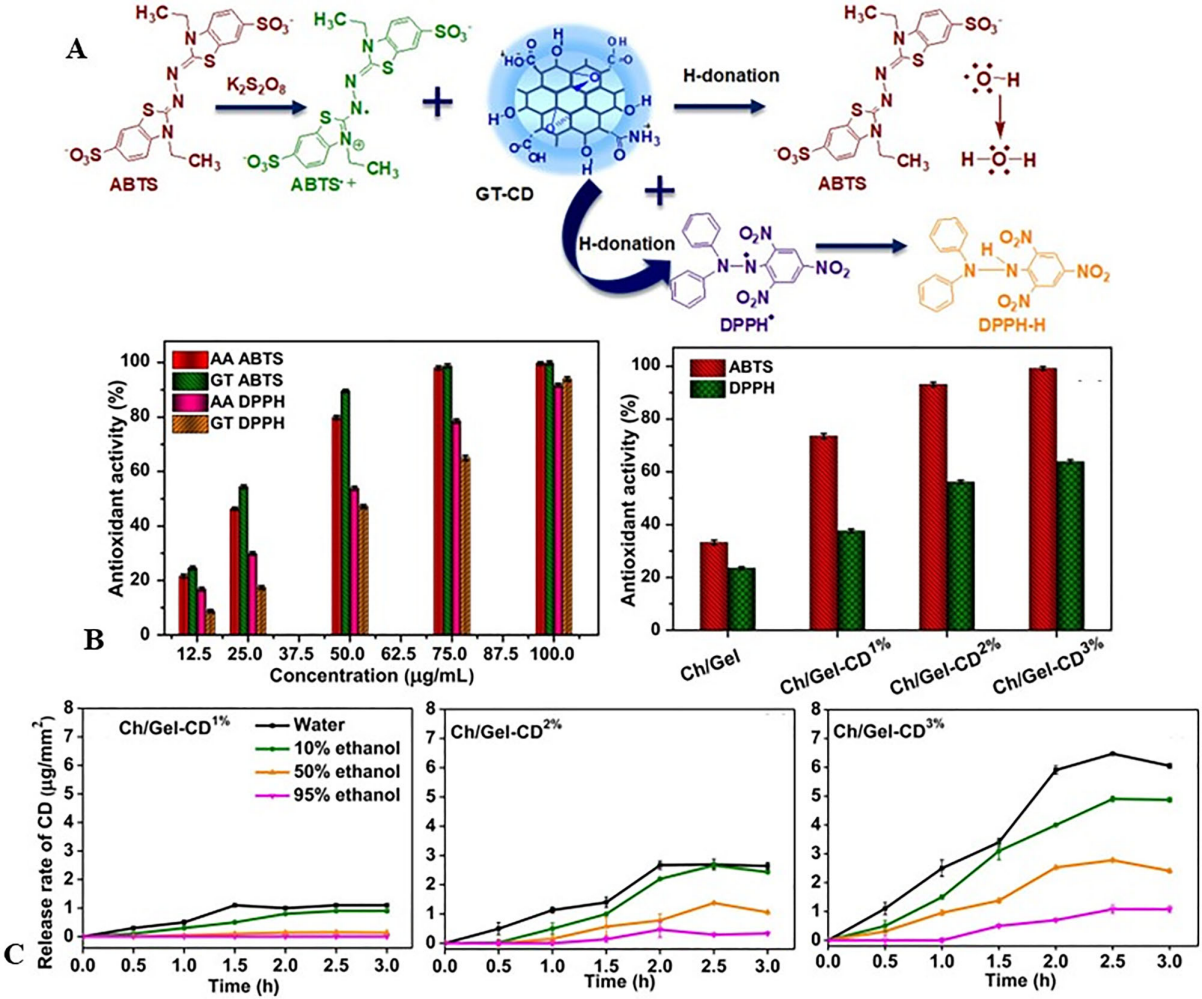
Proposed mechanism of radical scavenging activity of GT‐CD (A). Antioxidant activity of GT‐CD and chitosan/gelatin‐based films (B). Release profile of GT‐CD from chitosan/gelatin‐based films in various food simulants (C). Reprinted with permission (Khan, Ezati, et al. 2023a).

In meat preservation, CQDs are increasingly integrated into active packaging systems. Chitosan–starch films embedded with nitrogen‐ and phosphorus‐doped CQDs reduced *L. monocytogenes*, *E. coli*, and *S. aureus* populations to < 2.5 log CFU/g within 48 h while simultaneously blocking 93.1% of UV‐A and 99.7% of UV‐B radiation to prevent photooxidation (Khan, Ezati, et al. 2023b). Similarly, chitosan–gelatin films functionalized with green tea‐derived CQDs achieved complete inhibition of *E. coli* and *L. monocytogenes* within 3 h, maintaining bacterial counts below 1 log CFU/g for 24 h at 20°C (Khan, Ezati, et al. 2023a). As illustrated in Figure 9, GT‐CQDs exhibit dual antioxidant and antimicrobial functions: their radical‐scavenging activity neutralizes lipid oxidation byproducts, while controlled release in food simulants ensures sustained efficacy without migration risks.

Despite their promise, challenges remain in scaling CQD synthesis and navigating regulatory frameworks. Current research prioritizes green synthesis routes using food‐grade precursors (e.g., fruit peels and plant extracts) to improve cost‐effectiveness and sustainability. Further studies are also needed to elucidate long‐term safety profiles and optimize CQD–polymer interactions for industrial applications. By addressing these barriers, CQDs could revolutionize meat preservation, offering a safer, multifunctional alternative to conventional preservatives.

### 5.2 SiO_2_NPs

SiO_2_NPs have emerged as a cornerstone in active food packaging due to their unique porous structure, high biocompatibility, and capacity for controlled release of antimicrobial and antioxidant agents. These properties make them particularly effective in mitigating biological cross‐contamination and extending the shelf life of perishable meats. Their versatility is exemplified in diverse applications, ranging from nanocomposite films to synergistic systems combining nanotechnology with advanced processing techniques (Go et al. 2017; Lin, Peng, et al. 2023).

In a groundbreaking study, Ye et al. (2025) integrated mesoporous SiO_2_NPs loaded with d‐cysteine into PCL nanofibers and coupled this system with cold plasma dielectric barrier discharge technology. This hybrid approach achieved a 90% reduction in *E. coli* and *S. enteritidis* populations in pork, extending shelf life by 2 days while significantly suppressing lipid oxidation, a critical factor in preserving meat quality. The cold plasma enhanced nanoparticle dispersion and activation, demonstrating the potential of multi‐technology synergies for sustainable preservation.

Further innovations include cassava starch–CMC films functionalized with SiO_2_NPs and caffeic acid, developed by Lin, Peng, et al. (2023). These films exhibited 99.9% inhibition of *E. coli* and *S. aureus* in beef and chicken, attributed to the sustained release of caffeic acid from the silica matrix. The continuous antioxidant activity also reduced TBARS by 40%, highlighting SiO_2_NPs’ dual role in microbial control and oxidative stability.

SiO_2_NPs further enhance the performance of other nanomaterials, as demonstrated by Zhao et al. (2022), who embedded silica‐coated AgNPs into PVA films. This design achieved complete *E. coli* inactivation within 6 h in beef burgers, while the silica shell moderated silver ion release, prolonging antimicrobial efficacy during refrigerated storage. Although less effective against *S. aureus* due to its robust peptidoglycan layer, the system reduced bacterial loads by 85%, underscoring SiO_2_NPs’ utility in modulating nanomaterial reactivity.

Despite these advancements, challenges persist. The industrial scalability of SiO_2_NP‐based packaging requires optimization of synthesis methods to ensure cost‐effectiveness, particularly for mesoporous variants. Additionally, long‐term safety assessments are needed to address potential nanoparticle migration, especially in high‐fat meat matrices where silica interactions remain understudied. Future research should prioritize green synthesis routes and lifecycle analyses to align with regulatory and sustainability goals.

In conclusion, SiO_2_NPs represent a transformative tool in meat preservation, offering precise control over bioactive compound delivery. Their integration into smart packaging systems not only enhances food safety but also reduces reliance on high concentrations of synthetic preservatives, aligning with consumer demands for natural and minimally processed foods.

### 5.3 Nanocellulose

Nanocellulose, derived from plant or bacterial sources, has emerged as a sustainable and multifunctional material for meat preservation, offering exceptional mechanical strength, biocompatibility, and controlled release of bioactive compounds. Its versatility enables applications ranging from absorbent mats to nanocomposite films, effectively controlling pathogen proliferation while preserving sensory and nutritional quality. The inherent antimicrobial activity of nanocellulose arises from its high surface area, functionalizable hydroxyl groups, and capacity to disrupt microbial membranes, making it ideal for active packaging systems (Ahankari et al. 2021; Marchetti and Andrés 2021).

Tian et al. (2023) developed oxidized bacterial nanocellulose mats with high carboxyl group content (0.51 mmol/g) for chilled pork preservation. These mats inhibited *S. aureus* and *E. coli* by 92.4% and 85.2%, respectively, while reducing volatile basic nitrogen levels and stabilizing pH over 10 days at 4°C. The carboxyl groups facilitated electrostatic interactions with bacterial cells, enhancing antimicrobial efficacy without compromising meat texture. Similarly, Zabihollahi et al. (2020) engineered CMC films reinforced with cellulose nanofibers (CNFs; 35 nm diameter), which reduced *S. aureus*, *E. coli*, and *S. typhimurium* counts in chicken fillets through physical adsorption and prolonged release of natural antimicrobials.

The integration of cellulose nanocrystals (CNCs; 75 nm diameter) into chitosan films further demonstrated nanocellulose's adaptability (Costa et al. 2021). These composites exhibited fungicidal activity against *Candida albicans* and reduced volatile basic nitrogen by 30% in chicken meat, leveraging the synergistic effects of chitosan's cationic properties and CNC's barrier functionality. For lamb preservation, Khan, Li, et al. (2024) designed karaya gum films stabilized with bacterial nanocellulose (274.6 nm) and valerian root extract, achieving a 3‐log reduction in total viable counts over 9 days. The nanocellulose–Pickering emulsion system delayed lipid oxidation by 40% and protein degradation by 25%, maintaining sensory attributes such as juiciness and color.

Despite these advancements, challenges remain in scaling production and optimizing cost‐efficiency, particularly for bacterial nanocellulose. Future research should focus on green synthesis methods and hybrid systems combining nanocellulose with smart indicators for real‐time freshness monitoring. By addressing these barriers, nanocellulose can solidify its role as an eco‐friendly, high‐performance solution for next‐generation meat preservation.

## 6 Nanoencapsulation

Nanoencapsulation has become a pillar of innovation in meat preservation, addressing critical challenges associated with the instability and volatility of bioactive compounds. By entrapping antimicrobial and antioxidant agents—such as EOs (e.g., carvacrol, thymol), flavonoids (e.g., naringenin), and organic acids—within nanoscale carriers, this technology enables precise control over release kinetics, enhancing bioavailability and prolonging efficacy. Nanoencapsulation systems, including liposomes, polymeric nanoparticles, and solid lipid nanocarriers, mitigate limitations like hydrophobicity, oxidation, and sensory interference, ensuring targeted delivery while preserving meat's organoleptic properties. Furthermore, the integration of these systems into edible coatings, films, or absorbent pads synergizes with meat matrices, offering solutions to inhibit pathogen proliferation and oxidative rancidity. This section explores recent breakthroughs in nanoencapsulation design, their mechanistic roles in preservation, and their alignment with consumer‐driven demands for natural, residue‐free food safety interventions.

### 6.1 Nanoencapsulation of EOs

EOs are natural extracts derived from plants and are prized for their aromatic, therapeutic, and antimicrobial properties. Their bioactive constituents, including terpenes, phenols, ketones, and alcohols, exert antimicrobial effects by disrupting microbial cell membranes, inducing leakage of intracellular components, and impairing critical functions such as nutrient transport and ATP synthesis (Chouhan et al. 2017; Al‐Otaibi and AlMotwaa 2023). Despite their efficacy, EOs face industrial limitations due to volatility, oxidative instability, and hydrophobicity, which reduce their bioavailability in aqueous food matrices. Nanoencapsulation addresses these challenges by stabilizing EOs within nanocarriers, enabling controlled release and enhanced compatibility with meat systems (Chouhan et al. 2017).

Chitosan, a biopolymer with cationic functional groups, is a widely used nanocarrier for EO encapsulation (Hadidi et al. 2020; Abdelhady et al. 2023). Active chitosan–starch films reinforced with CNFs and clove essential oil (CEO) reduced *S. aureus* and *E. coli* counts by 3 log CFU/g in chilled beef over 5 days (Sreekanth et al. 2024). Similarly, sodium alginate coatings incorporating chitosan‐encapsulated CEO delayed lipid oxidation in pork by 50% while maintaining sensory quality for 15 days (M. Zhang et al. 2022). These systems leverage chitosan's electrostatic interactions with bacterial membranes and its ability to modulate EO release kinetics.

Hybrid nanoformulations combining EOs with metallic nanoparticles further enhance antimicrobial performance. Chitosan films functionalized with TiO_2_NPs and *Cymbopogon citratus* EO inhibited *Pseudomonas* spp. and *Enterobacteriaceae* in ground beef, extending shelf life by 10 days without compromising texture or color (Hosseinzadeh et al. 2020). Similarly, zirconium oxide nanoparticles and CuNPs, synergized with *Nigella sativa* EO in fish gelatin–chickpea protein films, achieved 99% inactivation of *E. coli* and *S. aureus* in ground meat (Sani et al. 2021; Rasul et al. 2022).

Innovative nanocarriers, such as MMT nanoclays and PLA, have expanded EO applications. Chitosan–MMT films loaded with ginger EO preserved beef microbiological and sensory quality for 15 days, while PLA–nanochitosan films with *Polylophium involucratum* EO extended lamb shelf life by reducing lipid oxidation and microbial counts (Y.‐P. Zhang et al. 2021; Tabatabaee Bafroee et al. 2020). Recent advancements include cellulose–gelatin nanofibers encapsulating black pepper EO, which delayed lipid oxidation in duck meat by 35%, and tea tree EO nanoemulsions that enhanced pork preservation through sustained antimicrobial action (Acharya et al. 2024; Cai et al. 2021).

**FIGURE 10:**
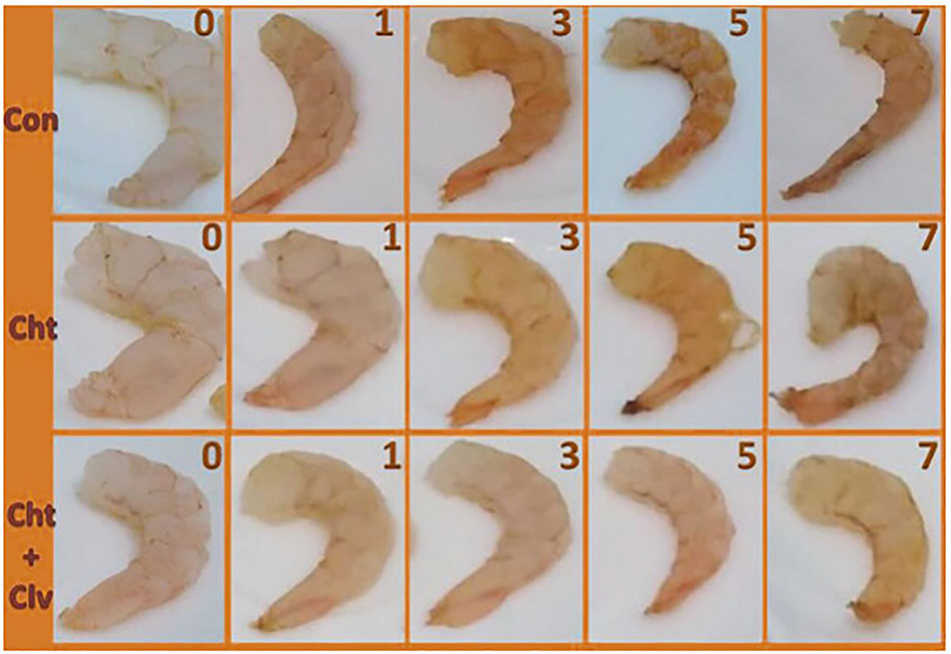
Effect of shrimp coating with 1.5% nanochitosan (Cht) and nanochitosan fortified with 1% clove extract (Cht + Clv) on the appearance and texture of samples compared to the control (Con) during refrigerated storage (4 ± 1°C) over 0, 1, 3, 5, and 7 days (Tayel et al. 2020). Reprint under the terms of the Creative Commons BY License.

Figure 10 illustrates the efficacy of nanochitosan–clove EO coatings in shrimp preservation, where treated samples maintained superior appearance, texture, and color over 7 days of refrigeration compared to controls (Tayel et al. 2020). This visual evidence underscores nanoencapsulation's role in mitigating spoilage‐driven quality degradation.

In conclusion, nanoencapsulation transforms EOs into robust tools for meat preservation, overcoming their inherent limitations while enhancing antimicrobial and antioxidant efficacy. By tailoring nanocarrier systems, from biopolymers to hybrid composites, researchers can optimize EO delivery, ensuring food safety and quality in alignment with consumer preferences for natural preservatives. Future efforts must prioritize scalable production methods, cost‐effectiveness, and comprehensive safety assessments to facilitate industrial adoption.

### 6.2 Other Nanoencapsulated Bioactive Compounds

Nanotechnology has revolutionized meat preservation by enabling the controlled release of bioactive compounds with antimicrobial and antioxidant properties, enhancing their efficacy while maintaining sensory quality. Nanoencapsulation stabilizes volatile or labile agents, prolonging their activity against pathogens and oxidative spoilage, which is critical for refrigerated storage where sustained protection is paramount (Q. Wang et al. 2020; Ab Rashid et al. 2022). Natural agents such as carvacrol, eugenol, thymol, flavonoids, and naringenin have emerged as key players, leveraging nanoencapsulation to overcome limitations like volatility and hydrophobicity, thereby preserving meat's organoleptic properties (Lin, Mei, et al. 2023; Q. Wang et al. 2020; Zaharioudakis et al. 2023; R. Zhang et al. 2023; da Silva et al. 2024).

**FIGURE 11:**
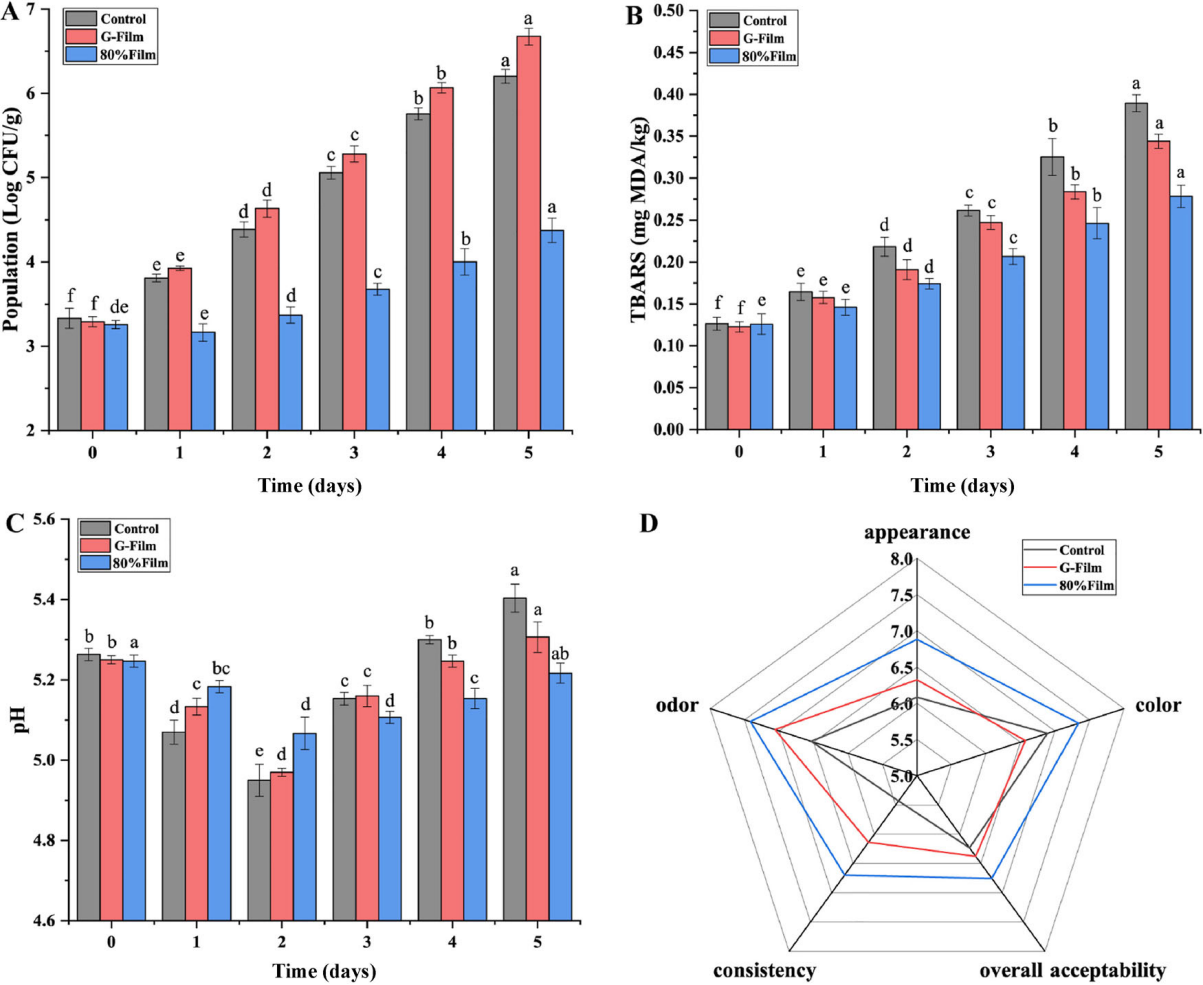
Variation in *S. aureus* population (A), TBARS values (B), and pH levels (C) in chicken breast stored at 4°C, alongside sensory evaluation results at the end of the storage period (D). Significant differences (*p* < 0.05) are indicated by letters. Reprinted with permission (Lin, Mei, et al. 2023). TBARS: thiobarbituric acid reactive substances.

Carvacrol, a phenolic monoterpene, disrupts microbial membranes and quorum sensing. Ovalbumin gel nanoparticles loaded with carvacrol reduced *Salmonella* populations in pork by 4 log CFU/g, demonstrating targeted delivery and prolonged efficacy (R. Zhang et al. 2023). Nanoemulsions of carvacrol outperformed microemulsions in ground pork, achieving a 3‐log reduction in *S. aureus* and *L. monocytogenes* while lowering TBARS, a lipid oxidation marker, by 35% (Zaharioudakis et al. 2023). Eugenol, encapsulated in gelatin–chitosan films, extended chicken breast shelf life by 10 days, maintaining pH stability and reducing TBARS by 50% compared to controls (Lin, Mei, et al. 2023; Q. Wang et al. 2020). Figure 11 illustrates these outcomes, showing suppressed *S. aureus* growth, stable pH, and superior sensory scores in coated samples.

Thymol nanoemulsions in chitosan coatings reduced *S. enteritidis* in ground chicken by 99% over 7 days, while limonene‐loaded chitosan nanoparticles (ChNPs) inhibited *E. coli* and *S. typhimurium* in ground meat by disrupting biofilms (da Silva et al. 2024; Badawy et al. 2020). Both systems delayed lipid oxidation by 40%, preserving color and texture without altering flavor profiles.

**FIGURE 12:**
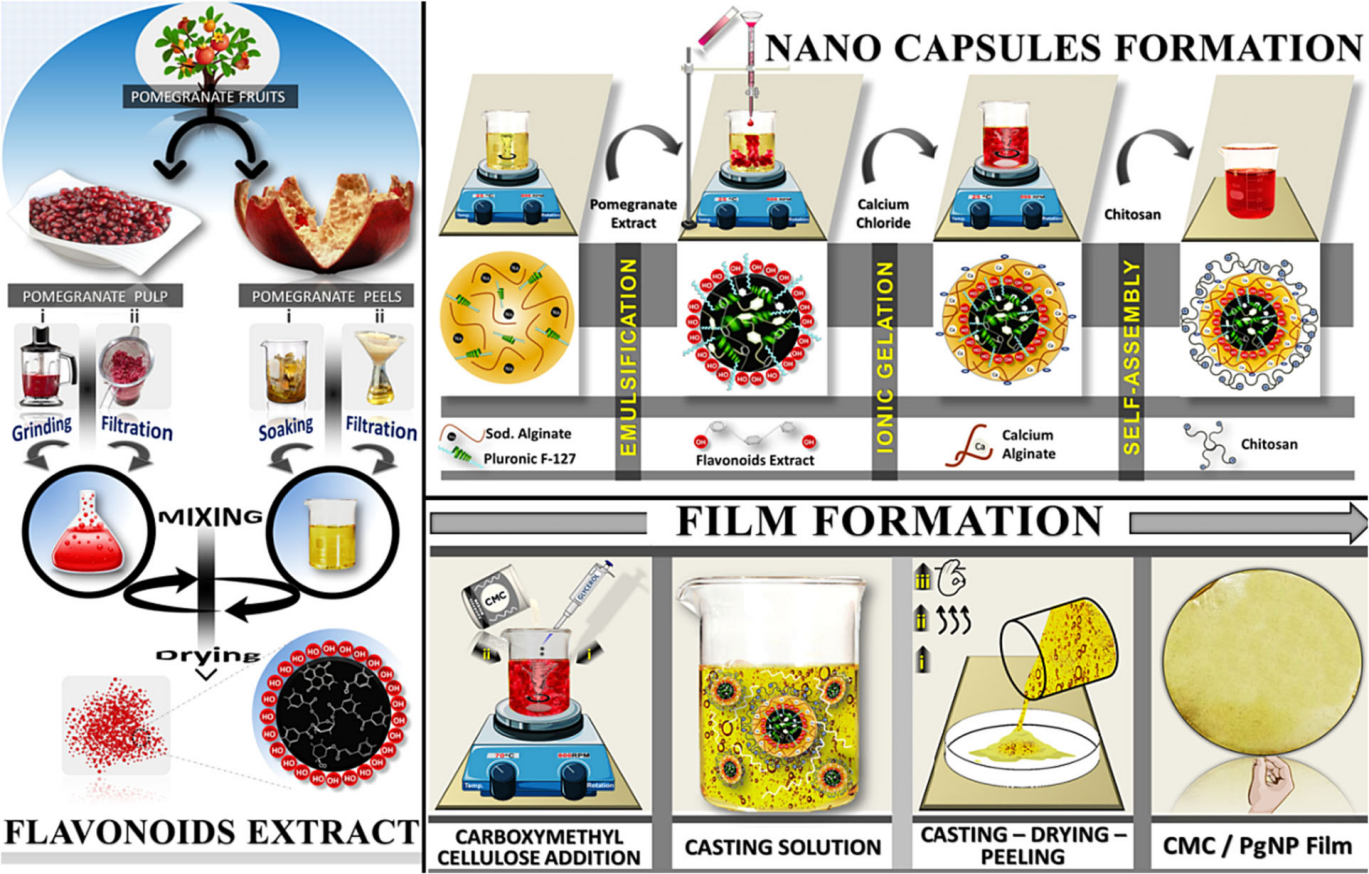
Experimental procedure for pomegranate (Pg) extraction, nanoencapsulation of flavonoids obtained from the extract, and development of the CMC‐based nanocomposite packaging film. Reprinted with permission (Khalid et al. 2024).

Pomegranate‐derived flavonoids, nanoencapsulated in CMC films, suppressed *C. jejuni* and *L. monocytogenes* in chicken and beef, extending shelf life by 7 days (Figure 12) (Khalid et al. 2024). Similarly, PVA‐encapsulated naringenin nanoparticles reduced oxidative rancidity in beef by 60%, maintaining microbiological and sensory quality for 5 days (Ab Rashid et al. 2022).

Nanoencapsulation enhances bioactive compounds through three primary mechanisms: targeted delivery, stabilization, and synergistic interactions. The cationic properties of chitosan, for instance, enable improved adhesion to negatively charged meat surfaces, ensuring localized release of antimicrobial agents while minimizing diffusion into the food matrix. Protein‐based carriers such as ovalbumin further stabilize bioactive compounds in lipid‐rich environments, shielding them from degradation and prolonging their activity. Hybrid systems, including flavonoid–metal composites, amplify antimicrobial and antioxidant effects by leveraging the complementary properties of individual components, creating a multifunctional defense against oxidative and microbial spoilage. Together, these mechanisms address critical challenges in meat preservation, such as lipid oxidation and pathogen proliferation, while maintaining sensory quality and extending shelf life through a tailored, multidimensional preservation strategy.

Therefore, nanoencapsulation transforms natural compounds into robust tools for meat preservation, balancing efficacy and sensory quality. By addressing scalability and safety, these innovations align with consumer demands for minimally processed, safer foods, paving the way for next‐generation preservation technologies.

## 7 Conclusion

The integration of nanotechnology into meat preservation represents a paradigm shift in combating biological cross‐contamination, with significant advancements demonstrated across multiple fronts. This review highlights the transformative potential of metallic nanoparticles, such as ZnONPs and AgNPs, which exhibit broad‐spectrum antimicrobial activity against key pathogens like *E. coli*, *Salmonella* spp., and *L. monocytogenes*, reducing microbial loads by 3–5 log CFU/g and extending shelf life by up to 28 days in refrigerated conditions. Nanoencapsulation systems, particularly those stabilizing volatile bioactive compounds like carvacrol, thymol, and flavonoids, have emerged as critical innovations, enabling controlled release and synergistic effects that preserve sensory attributes while delaying lipid oxidation by 35%–60%. The development of smart packaging, integrating nanomaterials with pH‐responsive indicators (e.g., anthocyanins) or moisture‐regulating nanocellulose, further underscores nanotechnology's dual role in real‐time spoilage monitoring and pathogen suppression.

These advancements address longstanding limitations of conventional methods, such as chemical residues and microbial resistance, aligning with consumer demands for minimally processed, “clean‐label” products. However, the translation of laboratory successes into industrial applications faces critical challenges. First, while nanomaterials like ZnONPs and AgNPs are widely studied, their long‐term toxicological profiles remain uncharacterized, particularly regarding nanoparticle migration into fatty tissues (e.g., silver accumulation exceeding 16.98 µg/mL in chicken meat). Second, regulatory frameworks lag behind technological innovation, as evidenced by the recent EFSA ban on titanium dioxide (E171) due to genotoxicity concerns. Third, scalability issues persist, with green synthesis methods for nanocellulose or CQDs requiring optimization for cost‐effective production. Additionally, environmental impacts, such as nanoparticle persistence in ecosystems, demand lifecycle assessments to ensure sustainability.

Future research must prioritize standardized protocols for nanoparticle safety evaluation, interdisciplinary studies to refine hybrid systems (e.g., SiO_2_NPs with cold plasma), and consumer acceptance trials to bridge the gap between innovation and market readiness. By addressing these limitations, nanotechnology can redefine meat preservation paradigms, offering safer, multifunctional solutions that enhance global food security without compromising quality.

## Author Contributions

**Camila Cristina Vieira Velloso**: writing–original draft, writing–review and editing, investigation. **Juliana Arriel Torres**: writing–review and editing. **Caue Ribeiro**: supervision, writing–review and editing, funding acquisition.

## Conflicts of Interest

The authors declare no conflicts of interest.

## Data Availability

Data will be made available on request.
